# Diffuse argyrophilic grain disease with TDP-43 proteinopathy and neuronal intermediate filament inclusion disease: FTLD with mixed tau, TDP-43 and FUS pathologies

**DOI:** 10.1186/s40478-023-01611-z

**Published:** 2023-07-06

**Authors:** Shunsuke Koga, Aya Murakami, Alexandra I. Soto-Beasley, Ronald L. Walton, Matthew C. Baker, Monica Castanedes-Casey, Keith A. Josephs, Owen A. Ross, Dennis W. Dickson

**Affiliations:** 1 Department of Neuroscience, Mayo Clinic, 4500 San Pablo Road, Jacksonville, FL 32224 USA; 2 Department of Neurology, Mayo Clinic, Rochester, MN 55905 USA; 3 Department of Clinical Genomics, Mayo Clinic, Jacksonville, FL 32224 USA

**Keywords:** NIFID, FUS, TDP-43, Argyrophilic grain disease, Frontotemporal lobar degeneration, Parkinsonism, LATE-NC

## Abstract

Frontotemporal lobar degeneration (FTLD) is a group of disorders characterized by degeneration of the frontal and temporal lobes, leading to progressive decline in language, behavior, and motor function. FTLD can be further subdivided into three main subtypes, FTLD-tau, FTLD-TDP and FTLD-FUS based which of the three major proteins – tau, TDP-43 or FUS – forms pathological inclusions in neurons and glia. In this report, we describe an 87-year-old woman with a 7-year history of cognitive decline, hand tremor and gait problems, who was thought to have Alzheimer’s disease. At autopsy, histopathological analysis revealed severe neuronal loss, gliosis and spongiosis in the medial temporal lobe, orbitofrontal cortex, cingulate gyrus, amygdala, basal forebrain, nucleus accumbens, caudate nucleus and anteromedial thalamus. Tau immunohistochemistry showed numerous argyrophilic grains, pretangles, thorn-shaped astrocytes, and ballooned neurons in the amygdala, hippocampus, parahippocampal gyrus, anteromedial thalamus, insular cortex, superior temporal gyrus and cingulate gyrus, consistent with diffuse argyrophilic grain disease (AGD). TDP-43 pathology in the form of small, dense, rounded neuronal cytoplasmic inclusion with few short dystrophic neurites was observed in the limbic regions, superior temporal gyrus, striatum and midbrain. No neuronal intranuclear inclusion was observed. Additionally, FUS-positive inclusions were observed in the dentate gyrus. Compact, eosinophilic intranuclear inclusions, so-called “cherry spots,” that were visible on histologic stains were immunopositive for α-internexin. Taken together, the patient had a mixed neurodegenerative disease with features of diffuse AGD, TDP-43 proteinopathy and neuronal intermediate filament inclusion disease. She met criteria for three subtypes of FTLD: FTLD-tau, FTLD-TDP and FTLD-FUS. Her amnestic symptoms that were suggestive of Alzheimer’s type dementia are best explained by diffuse AGD and medial temporal TDP-43 proteinopathy, and her motor symptoms were likely explained by neuronal loss and gliosis due to tau pathology in the substantia nigra. This case underscores the importance of considering multiple proteinopathies in the diagnosis of neurodegenerative diseases.

## Introduction

Frontotemporal lobar degeneration (FTLD) encompasses a group of disorders characterized by degeneration of the frontal and temporal lobes, leading to progressive decline in cognition, language, behavior and motor function [24]. FTLD can be classified based on the major protein accumulating in affected neurons and glia. The two most common subtypes are characterized by neuronal and glial inclusions composed of transactive response DNA binding protein of 43 kDa (TDP-43) and microtubule associated protein tau, referred to as FTLD-TDP and FTLD-tau, respectively [14, 24]. A third, less common subtype, is characterized by the abnormal accumulation of fused in sarcoma (FUS), FTLD-FUS [31]. FTLD-FUS can be further categorized into three entities: atypical FTLD with ubiquitin-positive inclusions, neuronal intermediate filament inclusion disease (NIFID), and basophilic inclusion body disease [14, 24].

The pathologic hallmarks of NIFID are eosinophilic, tau-negative and ubiquitin-negative inclusions in neurons and neuronal processes [5]. These inclusions are composed of neuronal intermediate filaments, including α-internexin [10]. NIFID primarily affects the frontal and medial temporal lobe, including the hippocampus and amygdala. Like other forms of FTLD, NIFID is associated with a range of clinical presentations, such as behavioral variant frontotemporal dementia (bvFTD), motor neuron disease, and atypical parkinsonian disorders [4, 9, 15, 27]. A recent study by Bieniek et al. demonstrated that NIFID is pathologically heterogeneous; NIFID is not always accompanied by FUS-positive inclusion bodies, and there are cases with and without striatal atrophy [4]. Consequently, it remains controversial whether NIFID should be considered a subtype of FTLD-FUS.

In this case report, we present an 87-year-old woman with a history of cognitive decline, gait problems, and hand tremors. Neuropathological evaluation revealed diffuse argyrophilic grain disease (AGD), atypical TDP-43 pathology and NIFID with FUS-positive inclusions, consistent with mixed FTLD (FTLD-tau, FTLD-TDP and FTLD-FUS). The simultaneous occurrence of three proteinopathies is rare, and such cases challenge clinical and neuropathological classifications of FTLD.

## Clinical summary

The patient was an 87-year-old woman who presented with at least a 7-year history of cognitive decline. At age 80 years, she complained of memory-related issues, but her daughter reported that she also had agitation, confusion, gait problems, hand tremors and balance issues. The latter caused her to lean towards one side and lose balance, requiring a wheeled walker for ambulation. She was taking Rivastigmine with unclear benefits and no side effects. She was not treated with anti-Parkinson’s disease medication. There was no known family history of neurologic disease. On neurological examination, she was able to name the month and year, but not the day or date. She required prompting to correctly identify her location. She had slightly decreased facial expression and mild resting tremor, but no cogwheel rigidity or focal weakness. With ambulation, she showed start hesitation and shuffling on turning and ataxia on tandem gait. An MRI of the brain revealed cerebral atrophy, disproportionate ventriculomegaly, and T2 signal in periventricular white matter. The patient was eventually placed in a nursing home, where she experienced recurrent deep vein thromboses treated with Coumadin. Her clinical diagnosis was Alzheimer’s type dementia.

## Materials and methods

### Neuropathological examination

An autopsy limited to an examination of the brain was performed after consent of the legal nextofkin. The left hemibrain was fixed in formalin and the right hemibrain was frozen. Regions sampled for histopathologic studies included six regions of the neocortex; two levels of the hippocampus; basal forebrain that includes the amygdala, lentiform nucleus and hypothalamus; anterior corpus striatum; thalamus at the level of the subthalamic nucleus; midbrain; pons; medulla; and two sections of the cerebellum, one including the deep nuclei. Paraffin-embedded 5-µm thick sections mounted on glass slides were stained with hematoxylin and eosin (H&E) and thioflavin S (Sigma-Aldrich, St. Louis, MO). Braak neurofibrillary tangle (NFT) stage and Thal amyloid phase were assigned by thioflavin S fluorescent microscopy according to published methods as previously described [34, 7, 26, 20]. Sections of the cortex, hippocampus, basal forebrain, brainstem and cerebellum were studied with immunocytochemistry for phosphorylated tau (CP13; mouse monoclonal; 1:1,000; gift from the late Dr. Peter Davies), phosphorylated TDP-43 (pS409/410; mouse monoclonal; 1:5,000; Cosmo Bio, Tokyo, Japan), FUS (rabbit polyclonal; 1:500; Proteintech, Rosemont, IL), α-internexin (rabbit polyclonal; 1:50; EnCor Biotechnology, Alachua, FL), and α-synuclein (EP1536Y; rabbit monoclonal; 1:40,000; Abcam, Waltham, MA), using IHC Autostainer 480S (Thermo Fisher Scientific Inc., Waltham, MA) and DAKO EnVision™ + reagents (Dako, Carpinteria, CA). Additionally, immunohistochemistry with anti-C9RANT (Rb5823; rabbit polyclonal; 1:1000, from Dr. Leonard Petrucelli, Mayo Clinic [2]) was performed in the cerebellum section. The severity of neuronal loss and gliosis, tau, TDP-43, FUS and α-internexin pathologies was graded semi-quantitatively on a four-point scale (0 = absent, 1 = mild, 2 = moderate, 3 = severe) on a multihead microscope by two investigators (SK and DWD).

### Immunofluorescence double-staining

Immunofluorescence double-staining was performed to study colocalization of TDP-43 and αinternexin. Deparaffinized and rehydrated sections were steamed in distilled water for 30 min, then blocked with Protein Block plus Serum Free (DAKO) for 1 h before incubation overnight at 4°C with antibodies to phospho-TDP43 (1:500) and α-internexin (1:50) diluted in with Antibody Diluent with Background-Reducing Components (DAKO). Sections were washed three times with buffered saline solution at room temperature, and then incubated with secondary antibodies Alexa Fluor 568 and Alexa Fluor 488 (1:500, Thermo Fisher Scientific, Inc.) diluted with Antibody Diluent with Background-Reducing Components (DAKO) for 1.5 h at room temperature in a dark chamber. Sections were washed three times with buffered saline solution at room temperature, incubated with 1% Sudan black for 2 min, washed with distilled water and mounted with Vectashield mounting media containing DAPI (Vector Laboratories). Representative images were taken with a confocal laser-scanning microscope (LSM 800; Carl Zeiss, Jena, Germany).

### Genetic analyses

For genotyping and whole genome sequencing, genomic DNA was extracted from frozen cerebellar tissue using standard procedures. *MAPT* sequencing was performed in exons 7, 9, 10, 11, 12, and 13, as well as known pathogenic intronic mutations located at 50 bp on either side of each exon (e.g., IVS10 + 16 C > T) as previously described [21]. Genotyping for *MAPT* H1/H2 (SNP rs1052553 A/G, A = H1, G = H2) was assessed with TaqMan SNP genotyping assays (Applied Biosystems, Foster City, CA). Samples were assessed with whole genome sequencing by the Mayo Clinic Genome Analysis Core (https://www.mayo.edu/research/core-resources/genome-analysis-core/services/sequencing). Variant call files generated by the Mayo Clinic Bioinformatics Core were annotated for genes associated with tauopathies, FTD and amyotrophic lateral sclerosis using Golden Helix SNP & Variation Suit v8.8.3 [18].

## Results

### Macroscopic findings

**Fig. 1:**
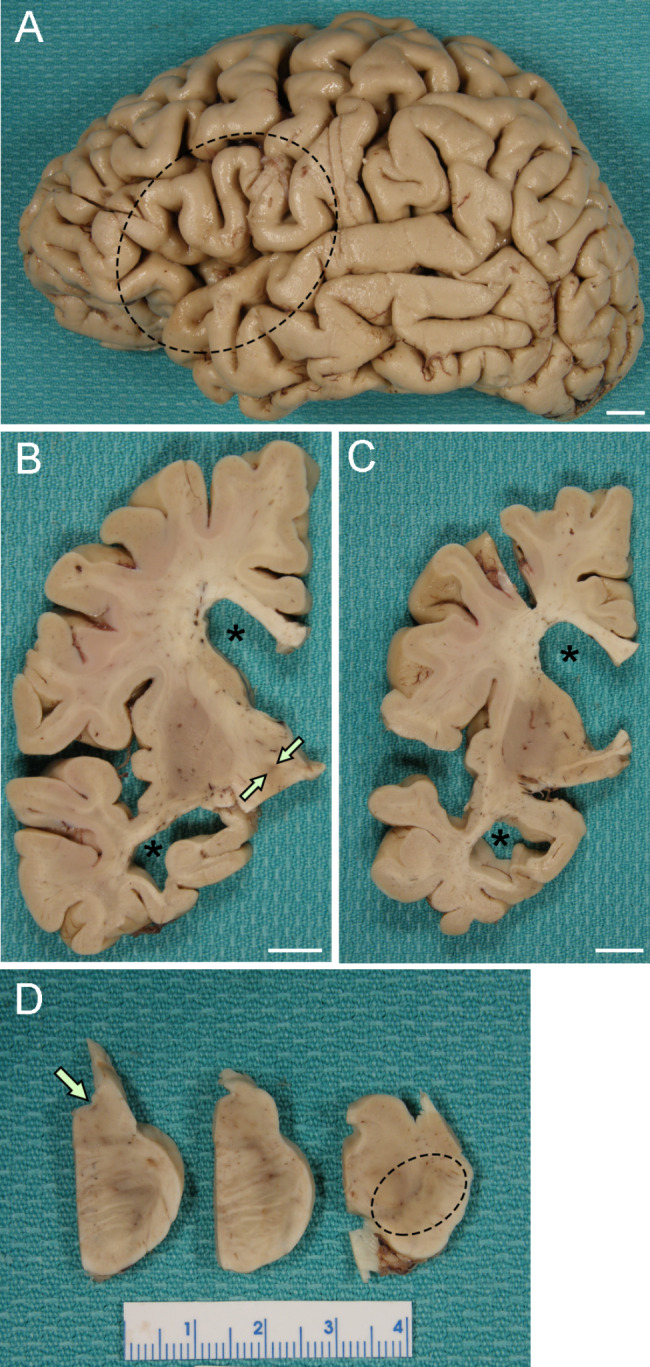
Representative macroscopic images. **(A)** Mild cortical atrophy is observed in the inferior frontal and superior temporal lobes (dotted circle). **(B and C)** The anterior and temporal horns are markedly dilated (asterisks), but the caudate nucleus is free of atrophy. The atrophy of the hippocampus (B) is observed, while the subthalamic nucleus (B, arrows) is preserved. **(D)** The locus coeruleus (arrow) and substantia nigra (dotted circle) show decreased pigmentation. Scale bars = 1 cm

At autopsy, the fixed left hemibrain weighed 480 g. Macroscopic findings revealed cortical atrophy in the frontal, temporal, parietal and occipital lobes (Fig. 1A). The medial temporal lobe also had mild atrophy. Sequential sections revealed marked enlargement of the anterior and temporal horns (Fig. 1B C). The cortical gray mantle was thinned, especially in the medial temporal lobe. The hippocampal formation and amygdala had marked atrophy. The subjacent white matter showed gray discoloration. The basal ganglia and the thalamus were macroscopically unremarkable. Horizontal sections of the midbrain, pons and medulla were free of obvious pathology. The substantia nigra and the locus ceruleus had decreased pigmentation (Fig. 1D). The cerebellar sections showed no unusual features.

### Histopathological findings

**Fig. 2:**
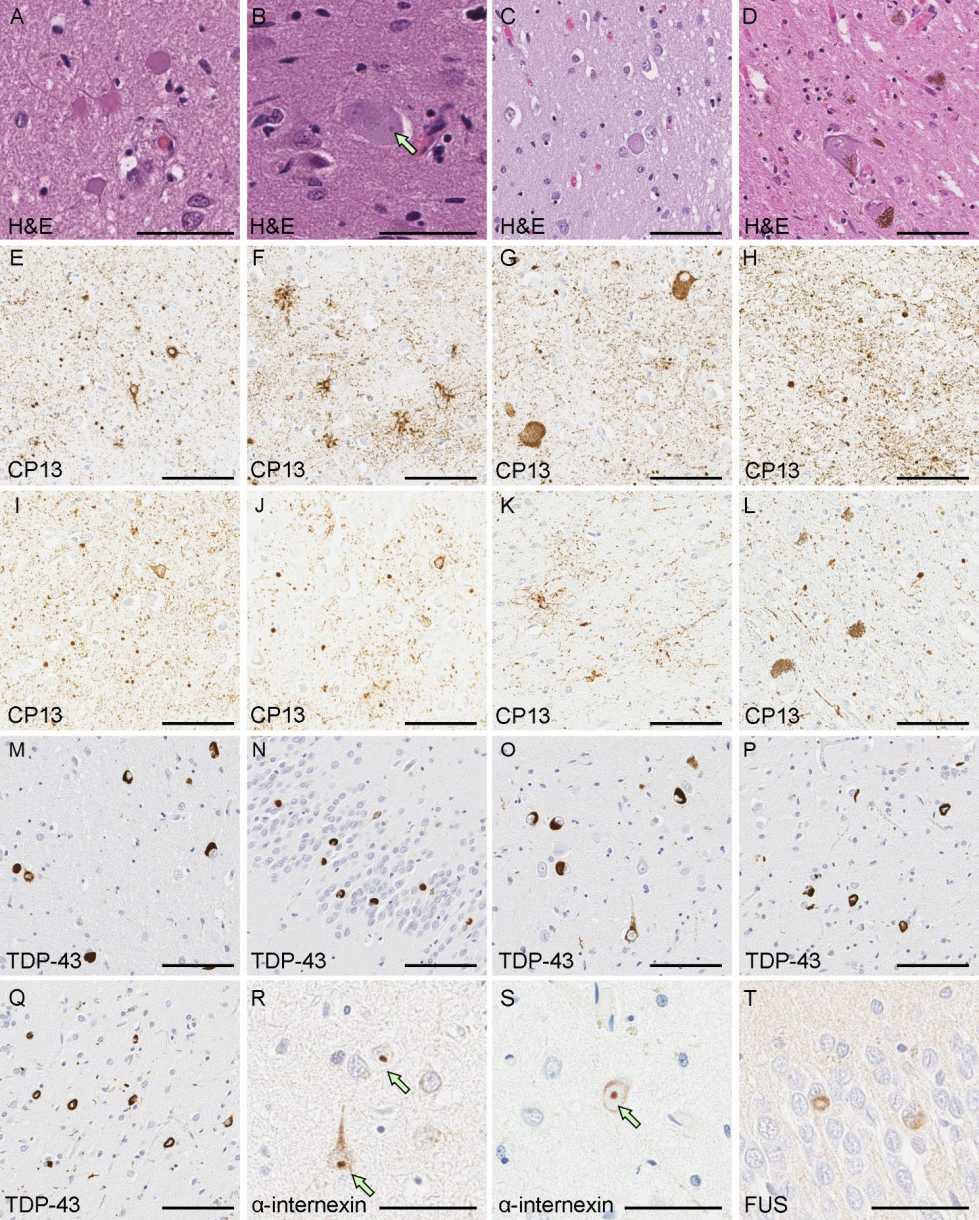
Representative images of H&E and immunohistochemistry. H&E staining shows severe neuronal loss with astrogliosis **(A)** in the amygdala a compact, eosinophilic inclusion in the subiculum **(B)**, and a balloon neuron in the insular cortex **(C)**. **(D)** The substantia nigra shows neuronal loss and gliosis with extracellular pigmentation. Immunohistochemistry for CP13 reveals argyrophilic grains (E), pretangles **(E)**, thorn-shaped astrocytes **(F)**, and balloon neurons **(G)** in the amygdala. The anteromedial thalamus has numerous threads and argyrophilic grains **(H)**. Argyrophilic grains and pretangles are observed in the insular cortex **(I)** and superior frontal gyrus **(J)**. **(K)** The midbrain tectum has granular/fuzzy astrocytes and thorn-shaped astrocytes. **(L)** Argyrophilic grains are also observed in the substantia nigra Immunohistochemistry for phospho-TDP-43 shows dense, compact neuronal cytoplasmic inclusions in the superior temporal gyrus **(M)**, dentate gyrus **(N)**, CA1 **(O)**, entorhinal cortex **(P)**, and occipitotemporal cortex **(Q)**. Immunohistochemistry for α-internexin reveals small neuronal intranuclear inclusions in the entorhinal cortex **(R)** and superior temporal gyrus **(S)**. Immunohistochemistry for FUS shows neuronal cytoplasmic inclusions in the dentate gyrus **(T)**. Scale bars: 50 μm in A, B, O and P; 100 μm in C-N.

The neocortex had atrophy, gliosis, neuronal loss and spongiosis in the medial temporal lobe, cingulate gyrus and orbitofrontal cortex, with better preservation the convexity gray matter in frontal, parietal and occipital lobes. The centrum semioval in frontal and temporal lobes had rarefaction. Severe neuronal loss and astrogliosis were observed in the amygdala (Fig. 2A), basal forebrain, nucleus accumbens, caudate nucleus and anteromedial thalamus. The hippocampus had neuronal loss and gliosis in the subiculum and in CA2 sector. A few compact neuronal eosinophilic inclusions (“cherry spots”) [15] were observed in the CA2 sector of hippocampus, subiculum (Fig. 2B), entorhinal cortex, amygdala and nucleus accumbens. Ballooned neurons were present in the cingulate gyrus, temporal lobe, insular cortex, claustrum and amygdala (Fig. 2C). The subthalamic nucleus, globus pallidus and putamen were relatively unremarkable. The substantia nigra had neuronal loss and gliosis with extraneuronal neuromelanin in ventrolateral and dorsomedial cell groups (Fig. 2D). The cerebellum showed well preserved Purkinje and internal granular cell layers. The cerebellar dentate nucleus was free of neuronal loss or grumose degeneration.

With thioflavin S fluorescence microscopy, senile plaques were not observed in the neocortices or hippocampus (Thal amyloid phase 0), and only a few NFT were detected in the entorhinal cortex and Sommer’s sector of hippocampus (Braak NFT stage III), consistent with primary age-related tauopathy [11]. There was no evidence of amyloid angiopathy in parenchymal or leptomeningeal vessels.

Phospho-tau immunohistochemistry revealed numerous argyrophilic grains, pretangles, tau-positive granular fuzzy astrocytes, thorn-shaped astrocytes, and ballooned neurons in the amygdala (Fig. 2E-G), consistent with AGD. This pathology extended to the hippocampus, parahippocampal gyrus, anteromedial thalamus (Fig. 2H), insular cortex (Fig. 2I), superior temporal gyrus, cingulate gyrus, superior frontal gyrus (Fig. 2J), midbrain tectum (Fig. 2K), and substantia nigra (Fig. 2L), consistent with AGD stage 3 or diffuse AGD [25, 33]. NFT, granular fuzzy astrocytes, and oligodendroglial coiled bodies were also found in the middle frontal gyrus (Table 1). Additionally, extensive ageing-related tau astrogliopathy with tau-positive thorn-shaped astrocytes was observed in the subpial and perivascular regions of the medial temporal lobe.

**Table 1:** The distribution and severity of pathology

Region	Neuronal loss & gliosis	pre-NFT & NFT	Coiled bodies	Tau^+^ astrocytes	Tau^+^ grains/threads	TDP-43 inclusions	FUS inclusions	α-internexin inclusions
Middle frontal gyrus	-	+	+	++	-	-	-	-
Superior frontal gyrus	+	++	+	+++	++	-	-	-
Cingulate gyrus	+	++	+	+++	+++	+	-	-
Motor cortex	-	-	-	+	-	-	-	-
Superior temporal gyrus	++	++	++	+++	+++	++	-	++
Caudate/putamen	-	++	+	+++	+++	+	-	-
Globus pallidus	-	+	+	+	++	-	-	-
Basal nucleus	-	++	+	-	++	-	-	-
Amygdala	++	+++	+	+	+++	+++	-	++
Dentate fascia	+	++	-	-	++	+++	+	+
CA1/subiculum	++	+++	-	-	+++	+++	-	+
Entorhinal cortex	++	+++	+	++	++	+++	-	+++
Insular cortex	+	++	+	++	+++	++	-	+
Hypothalamus	++	+++	+	++	+++	+	-	+
Anteromedial thalamus	++	+	+	-	+++	+	-	-
Red nucleus	-	-	-	+	+	-	-	-
Substantia nigra	++	+	-	-	++	-	-	-
Oculomotor complex	-	+	-	-	+	-	-	-
Midbrain tectum	+	+	+	+++	++	+	-	-
Locus ceruleus	+	+	-	+	+	-	-	-
Pontine tegmentum	-	+	-	++	++	-	-	-
Pontine base	-	-	-	-	-	-	-	-
Medullary tegmentum	-	+	-	-	+	-	-	-
Inferior olive	+	-	-	-	+	-	-	-
Dentate nucleus	-	-	-	-	+	-	-	-
Cerebellar white matter	-	-	+	-	-	-	-	-

Immunohistochemistry for phospho-TDP-43 showed many neuronal cytoplasmic inclusions (NCIs) in layers II and IV of the inferior and superior temporal gyri. The NCIs were dense and round or ring-shaped (Fig. 2M). A few short dystrophic neurites were present in layer II. No neuronal intranuclear inclusions were detected. No glial cytoplasmic inclusions were observed in the white matter. Dense, compact NCIs were frequent in the dentate gyrus (Fig. 2N), and many flame-shaped NCIs and short dystrophic neurites were present in the CA1 sector of hippocampus, subiculum, entorhinal cortex and amygdala (Fig. 2O-Q). Sparse perivascular glial inclusions were observed in the entorhinal cortex, but glial cytoplasmic inclusions were not observed in the white matter. Only a few NCIs were present in the midbrain tegmentum, while the middle frontal and inferior parietal gyri, as well as other brainstem regions, including the hypoglossal nucleus and inferior olivary nucleus, did not have TDP-43 pathology (Table 1).

**Fig. 3:**
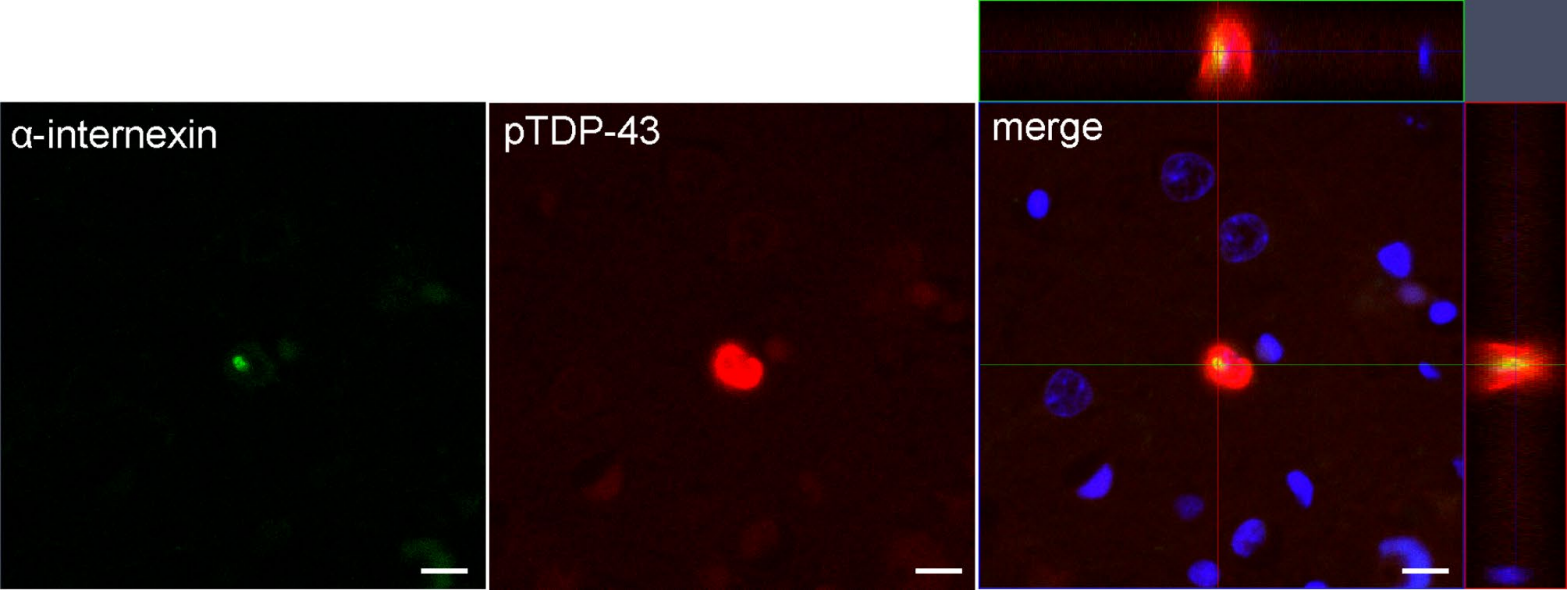
Immunofluorescence double-staining of α-internexin and phospho-TDP-43. α-internexin-positive neuronal intranuclear inclusion is observed in TDP-43-positive neuronal cytoplasmic inclusion in the entorhinal cortex. Scale bars: 10 μm

Immunohistochemistry for α-internexin showed neuronal intranuclear inclusions in the dentate gyrus, entorhinal cortex (Fig. 2R), amygdala, insular cortex, anterior hypothalamus, and superior temporal gyrus (Fig. 2S) (Table 1), consistent with NIFID. Immunohistochemistry for FUS revealed a few NCIs in the dentate fascia (Fig. 2T) and entorhinal cortex. Immunofluorescence double-staining revealed that some of the α-internexin-positive inclusions were in TDP-43-positive NCIs in the entorhinal cortex (Fig. 3). No colocalization of α-internexin and FUS was found in the dentate fascia and entorhinal cortex (data not shown). No C9RANT-immunoreactive inclusions [1] were detected in the cerebellum. Immunohistochemistry for α-synuclein did not reveal any Lewy-related pathology or glial cytoplasmic inclusion.

### Genetic analysis

We performed whole-genome sequencing using DNA extracted from frozen cerebellar tissue. No known mutations were detected in the genes associated with tauopathies and FTD/amyotrophic lateral sclerosis, including *MAPT*, *LRRK2*, *C9ORF72*, *ANG*, *ARGHEF28*, *CDH13*, *CHMP2B*, *FUS*, *GRN*, *HNRNPA1*, *PSEN1*, *PSEN2*, *SOD1*, *SQSTM1*, *TARDBP*, *TREM2*, *UBQLN2*, *VAPB*, and *VCP*.

## Discussion

In this case report, we present a patient with multiple neuropathological findings, including diffuse AGD, TDP-43 pathology, and NIFID. AGD and limbic predominant TDP-43 pathology are commonly observed in elderly individuals, while NIFID is a rare subtype of FTLD-FUS typically found in young patients. We consider diffuse AGD as the primary diagnosis in this patient based on the severity and extent of this pathology. For instance, there was severe neuronal loss and gliosis in the anteromedial thalamus, where tau pathology was also severe, but where αinternexin inclusions were minimal and TDP-43 and FUS inclusions were absent.

AGD is a 4-repeat tauopathy characterized by argyrophilic grains, pretangles, granular fuzzy astrocytes and ballooned achromatic neurons in the medial temporal lobe [6, 35]; however, a subset of AGD cases shows tau pathology that extends beyond the medial temporal lobe, involving the cingulate gyrus and superior temporal gyrus, which is referred to as diffuse AGD [25]. AGD often coexists with other neurodegenerative disorders, such as AD, progressive supranuclear palsy and corticobasal degeneration [12, 17, 39], which makes it challenging to establish clinicopathological correlations [40]. Patients with limbic AGD often present with amnestic mild cognitive impairment, dementia or psychiatric symptoms [12, 40], while those with diffuse AGD typically present with significant cognitive decline, including executive dysfunction, in addition to the amnestic features observed in patients with typical AGD. Despite the complicating concurrent neuropathologic findings (e.g., NIFID and TDP-43 pathology), her amnestic symptoms are likely, but not exclusively, related to her diffuse AGD [12].

Of note, our patient presented with motor symptoms suggestive of parkinsonism, in addition to dementia. Although an association between AGD and parkinsonism has not been fully investigated [13], Uchikado et al. reported a patient with limbic AGD who presented with dementia and parkinsonism [36]. Even without evidence of tau pathology or neuronal loss in the substantia nigra, the authors proposed that neuronal dysfunction could be present in AGD. Another study further supports this perspective by showing a reduction in dopamine content in the caudate nucleus and putamen in AGD [38]. Our patient had neuronal loss and gliosis in addition to tau pathology, but no TDP-43, α-internexin or α-synuclein pathology in the substantia nigra. Thus, diffuse AGD is the likely correlate of her parkinsonism.

TDP-43 pathology was also observed in the medial temporal lobe, brainstem and neocortex. The distribution and morphology of TDP-43 pathology (i.e., many NCIs and a few short dystrophic neurites) were consistent with FTLD-TDP type B [23], although characteristic granular NCI and glial cytoplasmic inclusions were absent. Limbic-predominant age-related TDP-43 encephalopathy neuropathological change (LATE-NC) can be considered because of the limbic-predominant distribution of TDP-43 pathology, her advanced age (87 years of age), and the lack of symptoms suggestive of FTD [3, 16, 29]. TDP-43 pathology in LATE-NC can extend beyond the limbic regions, even affecting the frontal cortex (LATE-NC Stage 3) [30]. The typical clinical presentations of LATE include slowly progressive amnesia that can range from mild cognitive impairment to dementia [29], as in this patient. It is notable, however, that genetically confirmed FTLD-TDP in old age also typically presents with amnestic dementia [8]. Therefore, we used the term TDP-43 proteinopathy to describe the pathology, rather than forcing the diagnosis into either FTLD-TDP or LATE-NC [28].

The presence of cherry spots on H&E staining raised the possibility of NIFID, prompting further immunohistochemistry for alpha-internexin and FUS. These additional analyses revealed α-internexin inclusions and FUS inclusions consistent with NIFID [10, 15], further complicating the neuropathological diagnosis. NIFID is a rare subtype of FTLD-FUS and is clinically and pathologically heterogeneous. Bieniek et al. reported that NIFID is not always FUS-positive and that it may not always be accompanied by striatal atrophy, which is a typical hallmark of FTLDFUS [4]. They reported one patient with co-occurrence of NIFID and TDP-43 pathology in a patient who did not have FUS or tau pathology. Patients with FUS-positive NIFID who have tau and TDP-43 pathology, as seen in this case, have never been reported, further highlighting the pathologic diversity of FTLD.

NIFID is often associated with the early age of symptomatic onset. Cairns et al. reported a mean age of onset of 40.8 years (range: 23–56) in a group of 10 patients [10], and Bieniek et al. reported a mean age of onset of 49.7 years (range: 37–64) in a group of 7 patients [4]. In contrast, our patient had late onset (80 years of age) dementia. Given the mixed pathology in our patient, weighing the relative importance of NIFID pathology to her clinical presentation is complicated by coexisting tau and TDP-43 pathologies. Her amnestic features might be better explained by AGD or TDP-43 pathology in medial temporal lobe structures.

Although the coexistence of AGD and FTLD-TDP or LATE-NC is common [19, 21, 37], the combination of three proteinopathies (i.e., tau, TDP-43, and FUS) is rare. Therefore, we performed whole genome sequencing to identify potential genetic causes of this condition; however, it did not reveal any known mutations in genes associated with tauopathies, FTD or amyotrophic lateral sclerosis. This was not completely unexpected given the late age of onset and her negative family history. AGD-like tau pathology has been reported in FTLD-tau due to *MAPT* S305I and S305S mutations [22, 32], but no mutation was observed in the *MAPT* gene.

Limitations of the present study include its retrospective nature, as the patient was enrolled in the State of Florida Alzheimer Disease Initiative, which does not include systematic and standardized longitudinal data collection. Consequently, clinical and neuroimaging information was limited, particularly in the late stages of the disease. As a result, it is possible that features consistent with bvFTD might not have been documented in the available medical records. Furthermore, it is uncertain whether her parkinsonism-like motor symptoms were responsive to levodopa treatment. Additionally, as this is a report of a single case, it is not possible to determine the frequency of our pathological findings. It remains unclear whether the observed co-occurrence of diffuse AGD, TDP-43 proteinopathy and NIFID was coincidental or whether there is a common mechanism for their concurrence. The whole genome sequencing analysis on this patient did not identify rare variants of pathogenic significance.

In conclusion, we present a patient with multiple neurodegenerative pathologies, including diffuse AGD, TDP-43 proteinopathy, NIFID and FUS pathology. The co-occurrence of these pathologies is rare, and such cases challenge a simple clinicopathologic diagnosis. We suggest that diffuse AGD is the primary diagnosis based on the severity and extent of pathology, while TDP-43 pathology may represent an unusual variant of LATE-NC. Our findings highlight the complexity and heterogeneity of clinical presentations in patients with multiple proteinopathies.

## Data Availability

The data that support the findings of this study are available from the corresponding author upon reasonable request.

## Abbreviations

AD: Alzheimer’s disease
AGD: Argyrophilic grain disease
bvFTD: Behavioral variant frontotemporal dementia
FTLD: Frontotemporal lobar degeneration
FUS: Fused in sarcoma
H&E: Hematoxylin and eosin
LATE-NC: Limbic-predominant age-related TDP-43 encephalopathy neuropathological change
NCI: Neuronal cytoplasmic inclusion
NFT: Neurofibrillary tangle
NIFID: Neuronal intermediate filament inclusion disease
TDP-43: Transactive response DNA-binding protein of 43 kDa;
