# Publisher Correction to: Diffuse argyrophilic grain disease with TDP-43 proteinopathy and neuronal intermediate filament inclusion disease: FTLD with mixed tau, TDP-43 and FUS pathologies

**DOI:** 10.1186/s40478-023-01621-x

**Published:** 2023-07-12

**Authors:** Shunsuke Koga, Aya Murakami, Alexandra I. Soto-Beasley, Ronald L. Walton, Matthew C. Baker, Monica Castanedes-Casey, Keith A. Josephs, Owen A. Ross, Dennis W. Dickson

**Affiliations:** 1 Department of Neuroscience, Mayo Clinic, 4500 San Pablo Road, Jacksonville, FL 32224 USA; 2 Department of Neurology, Mayo Clinic, Rochester, MN 55905 USA; 3 Department of Clinical Genomics, Mayo Clinic, Jacksonville, FL 32224 USA

**Publisher Correction to:** ***acta neuropathol commun *11, 109 (2023)**

10.1186/s40478-023-01611-z

Following publication of the original article [1], Supplementary Material was published which should not have been published since it was a copy of the source manuscript.

The publisher apologizes for the inconvenience caused.

The Supplementary Material has been removed; the original article [1] has been corrected.
